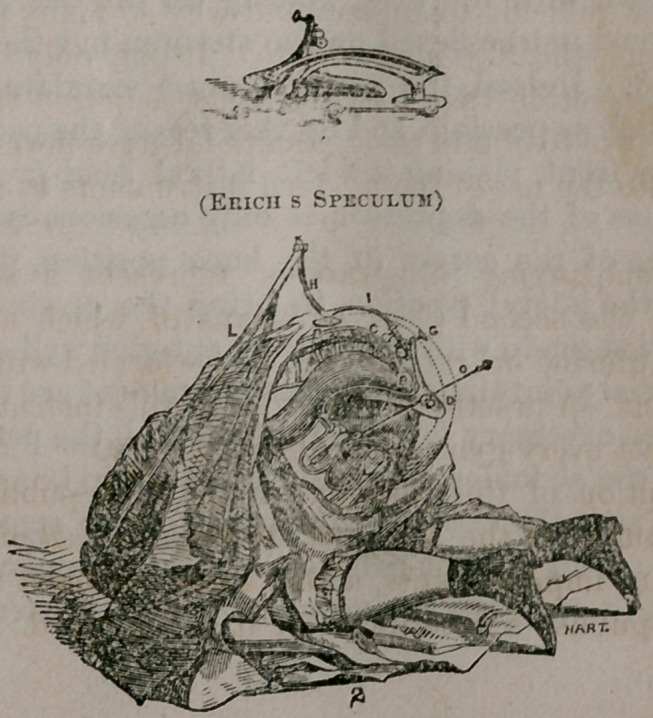# Self-Retaining Vaginal Speculum

**Published:** 1874-10

**Authors:** Augustus F. Erich

**Affiliations:** Baltimore, Md., Professor of Chemistry, College of Physicians and Surgeons


### Our Exchanges.

*BALTIMORE PHYSICIAN AND SURGEON—September:*

A SELF-RETAINING VAGINAL SPECULUM—*By Augustus F. Erich, M.D., of Baltimore, Md., Professor of Chemistry, College of Physicians and Surgeons.*—Vaginal specula can all be divided into two great classes: the old or tubular, and the modern, acting on the principle of Sims' speculum. To the first, or tubular class belong Ferguson's, Bozeman's, and all the different valvular specula.

The principal objections to this class are:

1. Their introduction is more difficult and often painful.

2. They push the uterus deeper into the pelvis, and do not permit the operator to draw it forward in a line with the axis of the vagina.

3. They do not expose simultaneously as much of the surface of the vagina, and compel us to operate through a contracted and unyielding orifice.

4. Not one of them will answer for all the different vaginal or uterine operations; thus necessitating the possession of a variety of specula.

The second or modern class, consisting of Sims' speculum and all its modifications, exposes the interior of the vagina by simply retracting the perineum, securing distension of the vaginal cavity by placing the patient in such a posture as will cause the pelvic organs to gravitate from the retractor. They are not open to the above objections, and are so much superior for all purposes that it seems difficult to understand why so many practitioners are still using the old instruments. It can only be explained upon the supposition that they have not acquired the necessary skill in their use to obtain good results. I have never known one accustomed to the use of the modern instruments to revert to the old ones.

The accompanying illustrations represent a self-retaining speculum of the second class, by means of which absolutely all vaginal and uterine operations may be performed without the aid of an assistant. A practitioner possessing this instrument is fully armed to meet every gynæcological emergency.

A description of this instrument was first published in the February number of the *New York Medical Journal* of 1869. Several important improvements, added since, render it necessary at this time to publish a description of it as improved.

Fig. 1. represents the speculum folded up in its most portable shape.

Fig. 2. represents it as used with the patient upon her knees, being the most favorable position for the purpose of showing all its parts. It can, however, be used with equal facility in the left lateral semi-prone position.

Before the speculum is applied the patient ought to loosen the fastenings of such portions of her dress as may compress her abdomen, then put her head and right arm through the loop of the strap and assume the position represented in the woodcut, her back curved downward, her knees to be separated about eight or ten inches, and her thighs to be at right angles with the table upon which she is kneeling. Now grease the retractor and introduce it upon the index-finger of the right hand, pushing it gently but firmly up as far as possible, press the tip of the retractor with the finger toward the sacrum as far as the posterior wall of the vagina will yield to gentle pressure, and use the screw *G* to secure it in the position thus attained. Then draw the ascending lever *H* upward until the perineum is sufficiently retracted, and fix it in that position by attaching the strap to the hooks at the upper end of the lever *K*. Should the patient suffer from lateral version, the instrument can be adjusted to any desired lateral angle by lightly loosening the screw *C;* and should there be any tendency to other displacements of the uterus, these may be adjusted and the uterus fixed in any desired position by means of the depressor.

(Erich s Speculum)

2

*A*—the speculum or retractor, the right wing of which is longer than the left, to support the right buttock when the patient is in the left lateral semi-prone position.

*O*—a depressor, having a sliding as well as a circular motion. It may be securely fixed in any desired position by simply tightening the screw *D*, by means of which it is attached to the speculum. A few turns of the same screw in the opposite direction will detach the depressor and washer in which it slides, leaving the opening of the speculum unobstructed. The depressor may then be used with the hand, as an ordinary one.

*B*—a screw, by means of which the speculum can be detached and others of different sizes secured to the levers.

*G*—a screw, by which the antero-posterior angle of the speculum may be adjusted.

*C*—a screw, by means of which the speculum may be fixed at any desired lateral angle.

*H*—the ascending lever, with a row of steel hooks at its upper extremity for the attachment of the strap.

*L*—a strap passing under the right axilla and over the left shoulder, the united ends of which are attached to the lever *H*, securely fixing it at any degree of tension.

To use the speculum in the left lateral semi-prone position, the same directions should be followed, excepting those relating to the position of the patient, which is described by Sims as follows: "The patient is to lie on her left side. The thighs are to be flexed at about right angles with the pelvis, the right being drawn up a little more than the left. The left arm is thrown behind across the back, and the chest rotated forward, bringing the sternum very nearly in contact with the table, while the spine is fully extended, with the head resting on the left parietal bone. The head must not be flexed on the sternum nor the right shoulder elevated. Indeed, the position must simulate that on the knees as much as possible, and for this reason the patient is rolled over on the front, making it a left lateral semi-prone position." While the use of the depressor is only occasionally required for the exposure of the cervix in the knee position, it is generally needed in the lateral position, to bring the cervix into view by gentle traction made with it upon the anterior wall of the vagina.

The special points in favor of this instrument are the following:

The force necessary for the retraction of the perineum is exerted upon the shoulder, not against the sharp bony edge of the pubic arch, as is the case in a number of other specula. It can, therefore, be used without pain in the most muscular subject, and enables us to retract the perineum to its fullest extent.

It leaves the orifice of the vagina as free of all obstructions as the simple Sims' speculum, and has therefore been frequently used to facilitate the introduction of pessaries.

It follows all the motions of the patient, never losing its relative position to her body. In consequence of which it is even superior to Sims' speculum when held by a skilful assistant, especially during tedious operations, when it frequently becomes necessary for the operator to put down his instrument and correct the position of the speculum with his own hands, because he finds it next to impossible to convey to the assistant a description of the required position by any other means; this difficulty being unavoidable while the speculum is supported by the hands of an assistant, and consequently liable to move independently of the patient's body, and while the assistant cannot be in such a position as to enable him to see whether the speculum is in its proper position or not.

## Figures and Tables

**Figure f1:**